# Contribution of anti-inflammatory and anti-virulence effects of azithromycin in the treatment of experimental *Staphylococcus aureus* keratitis

**DOI:** 10.1186/s12886-020-01358-4

**Published:** 2020-03-06

**Authors:** Kana Ikemoto, Shinya Kobayashi, Yu Haranosono, Seiko Kozai, Tomoyuki Wada, Hideki Tokushige, Akio Kawamura

**Affiliations:** Senju Pharmaceutical Co., Ltd., 6-4-3 Minatojima-Minamimachi, Chuo-Ku, Kobe, Hyogo 650-0047 Japan

**Keywords:** Anti-inflammatory, Anti-virulence, Azithromycin, Keratitis, *Staphylococcus aureus*, Ocular infection

## Abstract

**Background:**

We aimed to demonstrate the contribution of anti-inflammatory and anti-virulence effects of azithromycin (AZM) in ocular surface infection treatment.

**Methods:**

*Staphylococcus aureus* was injected into the corneal stroma of rabbits to induce keratitis. AZM at concentrations of 0.01, 0.1, and 1% was instilled into the eye twice daily. The eyes were examined using a slit lamp and scored. The viable bacteria in the cornea were counted at 48 h post infection. To evaluate the anti-inflammatory efficacy of AZM, *S. aureus* culture supernatant-induced anterior ocular inflammation in rabbit was examined using a slit lamp and scored. To evaluate the inhibitory effect of AZM on bacterial toxin production, *S. aureus* was cultured with AZM and hemolytic reaction in the culture supernatant was determined.

**Results:**

In the bacterial keratitis model, AZM dose-dependently inhibited the increase in the clinical score. The viable bacterial count in the cornea treated with 1% AZM significantly decreased compared with that of the vehicle, whereas bacterial count in 0.01 and 0.1% AZM-treated corneas was similar to that of the vehicle. In the anterior ocular inflammation model, 0.1 and 1% AZM inhibited the increase in the clinical score. AZM inhibited hemolytic reaction at concentrations that did not inhibit bacterial growth.

**Conclusions:**

The results demonstrated that AZM has not only anti-bacterial, but also anti-inflammatory effects, and inhibits bacterial toxin production leading to ocular surface damage in bacterial infection. Thus, the therapeutic effect of AZM against ocular infections is expected to be higher than that which could be assumed if it only had anti-bacterial activity.

## Background

After bacterial invasion, the initial acute inflammatory response against the bacteria is initiated by the innate immune system of the body to eliminate the pathogen and further stimulate adaptive immune responses [1]. Although these processes are important to eradicate the invading pathogens, they have the risk of simultaneously damaging the host tissues. Furthermore, the ability of bacteria to cause diseases is associated with virulence factors such as leukocidal and hemolytic toxins, which are known to induce inflammation [2, 3]. Ocular infectious diseases can cause scarring due to the inflammatory response as describe above, which may not be easily resolved, and ultimately result in blindness or visual impairments [4]. This encourages the use of broad-spectrum bactericidal agents for the treatment of ocular infections without identifying causative pathogens or without testing their susceptibility to anti-microbial agents. Anti-virulence therapy, which is independent of bacterial growth, can be a potential treatment option for ocular infection. Several studies have demonstrated that inhibition of bacterial toxin production could suppress ocular tissue damage [5, 6].

Azithromycin (AZM) is a broad-spectrum antibiotic that has been shown to be effective against gram-positive, gram-negative, and atypical bacteria. It is widely used in clinical practice. It has been recognized that AZM exerts not only anti-bacterial activity but also anti-inflammatory effects, which are related to the NF-κB pathway [7–10], inhibition of quorum sensing, and anti-virulence effects [11–14]. The anti-inflammatory effect of AZM in the ocular surface has been demonstrated in lipopolysaccharide-induced rats and in corneal epithelial cells stimulated with zymosan [9, 15]. These studies have shown that AZM suppresses the signs of inflammation and reduces the level of several proinflammatory factors. Although the anti-virulence effect of AZM on gram-positive bacteria which are the major causatives of ocular infection is poorly known, it is well known that AZM suppresses the production of bacterial virulence factor and formation of biofilm via inhibition of quorum sensing in *Pseudomonas aeruginosa* [11, 12]. These studies provide evidence of other effects besides the conventional anti-bacterial effect of AZM, but it is unclear whether anti-inflammatory and anti-virulence effects of AZM can contribute to the treatment of ocular infection.

In this study, we investigated whether AZM exerts anti-inflammatory and anti-virulence effects in addition to anti-bacterial activity in a staphylococcal keratitis model in order to elucidate their contribution in the treatment of ocular infection.

## Methods

### Reagents

Azithromycin at concentrations of 0.01, 0.1, and 1% along with its vehicle was prepared for this study by Senju Pharmaceutical Co., Ltd. (Osaka, Japan). These formulations all contained DuraSite®, which is a proprietary polymeric mucoadhesive delivery system, prepared by Senju Pharmaceutical Co., Ltd. An ophthalmic corticosteroid, betamethasone sodium phosphate 0.1% (Rinderon® ophthalmic, otic, and nasal solution 0.1%), was purchased from Shionogi & Co., Ltd. (Osaka, Japan). Saline (Otsuka normal saline) was purchased from Otsuka Pharmaceutical Factory, Inc. (Tokushima, Japan).

Ten milligrams of azithromycin dihydrate, which was purchased from Tokyo Chemical Industry Co., Ltd. (Tokyo, Japan) for in vitro assays, was dissolved in 1 mL of ethanol. That solution was then filtered and diluted with bacterial growth medium. Preserved rabbit blood was purchased from Kojin Bio Co., Ltd. (Tokyo, Japan).

### Animals

Seventy-three male Japanese white rabbits weighing 1.3–1.9 kg were supplied by Kitayama Labes Co., Ltd. (Nagano, Japan). All procedures were performed in accordance with the statement of the Association for Research in Vision and Ophthalmology and the guidelines for animal experimentation of Senju Pharmaceutical Co., Ltd., and the protocol was approved by the Institutional Animal Care and Use Committee (approval nos. 20,140,903–01, and 20,160,310–01).

### Bacterial strain

*Staphylococcus aureus* ATCC 25923 supplied by the American Type Culture Collection (Manassas, VA) was used in this study. The minimal inhibitory concentrations of AZM was reported to be 2 μg/mL for this strain, and it was defined as AZM-susceptible based on the Clinical Laboratory Standards Institute breakpoint (M100-S18) [16]. Furthermore, it has been reported that this strain can induce keratitis in rabbits [17, 18]. *Staphylococcus aureus* was grown at 32 °C on soybean casein digest (SCD) agar (Nihon Pharmaceutical Co., Ltd., Tokyo, Japan) or tryptic soy broth (TSB) (Becton, Dickinson and Company, Franklin Lakes, NJ).

### *S. aureus* keratitis rabbit model

Forty-one rabbits were anesthetized by intramuscular injection of a 3:1 mixture of 50 mg/mL ketamine (Ketalar® for intramuscular injection 500 mg; Daiichi-Sankyo Propharma Co., Ltd., Tokyo, Japan) and 20 mg/mL xylazine (Celactar® 2% injection; Bayer Yakuhin, Ltd., Tokyo, Japan). *Staphylococcus aureus* cells were suspended with saline. The viable bacteria were quantified using the agar dilution plate method (4.7 × 10^6^ colony-forming units (CFU) per milliliter). A 30 μL of bacterial suspension was injected into the corneal stroma of right eye. The left eye was not used for throughout the experiment. To avoid that the shortage of the number of viable bacteria limited the assessable dose-response range, the number was set higher than those of the previous reports. Oxybuprocaine hydrochloride ophthalmic solution (Benoxil® ophthalmic solution 0.4%; Santen Pharmaceutical Co., Ltd., Osaka, Japan) was topically applied prior to inoculation. Slit-lamp examination (SLE) was performed at 5, 12, 24, 36, and 48 h after inoculation, and the clinical severity of ocular discharge, hypopyon, and infection signs in the cornea, conjunctiva, nictitating membrane, and iris were scored according to a previously reported standard scale of ocular infection [19]. A masked observer performed all SLE scoring. Eleven rabbits were excluded due to leakage of inoculated suspension from corneal stroma immediately after administration. Thirty rabbits that were successfully inoculation were selected and randomly divided into four groups (*n* = 7–8). Azithromycin at concentrations of 0.01, 0.1, and 1% or its vehicle was instilled into the right eye (50 μL/eye, twice daily for 2 days). The topical treatment was started 5 h post inoculation when animals presented sign of ocular infection. The rabbits were euthanized by an intravenous overdose of sodium pentobarbital after SLE at 48 h. The corneas were removed, finely minced, serially diluted with sterile saline, and plated onto mannitol salt agar plates for quantification of viable bacteria in the cornea.

### Bacterial culture supernatant-induced anterior ocular inflammation rabbit model

Bacterial cells were grown overnight in TSB, centrifuged, and passed through a 0.22-μm filter to obtain bacterium-free *S. aureus* supernatant (SaS). Thirty-two rabbits were randomly divided into four groups. In total, 50 μL of SaS was instilled into the rabbit’s right eye three times at 10 min intervals to induce anterior ocular inflammation. Physiological saline or AZM at concentrations of 0.1 and 1% was instilled at 2 h before and at 5 h after the first challenge (50 μL/eye, twice daily). Betamethasone sodium phosphate 0.1%, was instilled into the eye for 2 days from the day before the induction of inflammation (50 μL/eye, 4 times daily). This ophthalmic corticosteroid was used as a positive control to elucidate the effectiveness of anti-inflammatory treatment in our model. SLE was performed at 3, 6, 12, and 24 h after the first challenge, and the severity was scored according to the criteria used for the keratitis model.

### Assay of hemolytic reaction

Bacterial cells were grown in TSB with or without (control group) AZM at concentrations of 0.008–8 μg/mL for approximately 16 h under agitation. After measuring the optical density at 660 nm to evaluate the growth of bacterial cells in the culture solution, bacterial culture supernatant was collected by centrifugation at 1610×*g* for 2 min and the residual bacterial cells were removed using a 0.22-μm filter. The bacterial supernatant was incubated with equal volume of 1% rabbit red blood cells suspended in saline for 10 min at 37 °C. The reaction mixture was centrifuged at 1000×*g* for 10 min to remove the intact red blood cells, and the supernatant was transferred into a 96-well microplate. Hemolysis was detected by measuring the absorbance at 440 nm as an indicator of hemoglobin release into the supernatant. The absorbance of the control group was defined as 100% and the bacterial number and the hemolysis rate of each group were calculated.

### Statistics

Differences in the SLE scores and CFU per cornea in the bacterial keratitis model between the AZM-treated and vehicle groups were compared using the one-sided Shirley–Williams test and one-sided Williams test, respectively. Differences in the SLE scores in SaS-induced anterior ocular inflammation model between each drug group and saline group were compared at each time point using the one-sided Steel test. Differences in the rate of hemolysis and bacterial number in the in vitro assay between the AZM-contained and control groups were compared using the one-sided Williams test. The results with a *p* value of < 0.05 were considered statistically significant. Data were processed and analyzed using Microsoft Excel 2010 (Microsoft Corp.) and Ekuseru–Toukei 2012 (Social Survey Research Information Co., Ltd.).

## Results

### Effect of AZM on bacterial keratitis model

**Fig. 1:**
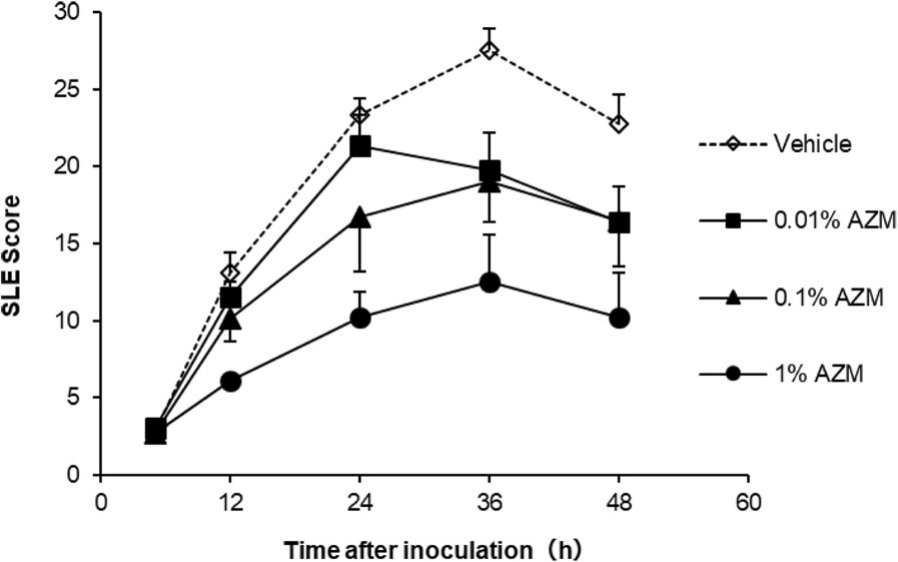
Effect of 0.01, 0.1, and 1% azithromycin in the rabbit bacterial keratitis model induced by intrastromal injection of 1.41 × 10^5^ CFU of *S. aureus.* The data represent the mean and standard error (*n* = 7–8)

Inoculation of *S. aureus* into the corneal stroma in rabbits induced clinical signs mainly in the cornea, conjunctiva, nictitating membrane, and iris. In the vehicle group, the SLE score was increased at 5 h after inoculation and reached the maximum at 36 h after inoculation (Fig. 1, Table 1). Azithromycin decreased the SLE scores in a dose-dependent manner. The SLE score in the 0.01 and 0.1% AZM groups reached the maximum at 24 and 36 h, respectively, and they were significantly lower than that of the vehicle group at 36 h after inoculation (*p* < 0.05; Table 1). The SLE score of the 1% AZM group reached the maximum at 36 h after inoculation, and the scores were significantly lower than those of the vehicle group from 12 to 48 h after inoculation. Treatment with 1% AZM significantly decreased the number of viable bacteria per cornea compared with that of the vehicle (*p* < 0.05), although the numbers of viable bacteria per cornea in the 0.01 and 0.1% AZM groups were similar to that of the vehicle group (Table 2).

**Table 1:** Median of slit lamp examination score in the rabbit bacterial keratitis model

Treatment		n	Median (1st quartile – 3rd quartile)				
			Time after inoculation				
			5 h	12 h	24 h	36 h	48 h
Vehicle		7	2.50 (2.25–2.75)	13.00 (11.00–15.00)	23.00 (21.25–25.00)	29.00 (25.50–29.75)	25.50 (18.50–25.75)
Azithromycin	0.01%	8	3.50 (2.63–4.00)	12.25 (9.38–13.25)	21.50 (18.75–25.00)	21.00 * (15.75–24.63)	18.25 (15.88–19.88)
	0.1%	7	3.00 (2.00–3.00)	11.00 (7.50–13.00)	21.50 (8.25–23.25)	21.50 * (13.00–24.50)	22.00 (10.25–22.75)
	1%	8	2.25 (2.00–3.50)	6.00 * (5.50–6.50)	10.25 * (7.75–12.88)	11.75 * (6.88–16.63)	9.50 * (6.38–12.00)

*p < 0.05 compared with the vehicle (Shirley–Williams test, one-sided)

**Table 2:** Number of viable bacteria per cornea at 48 h post-inoculation

Treatment		n	Mean ± S.E.M (log_10_ CFU/cornea)
Vehicle		7	3.64 ± 0.51
Azithromycin	0.01%	8	3.12 ± 0.41
	0.1%	7	3.70 ± 0.46
	1%	8	2.12 ± 0.29 *

The detection limit was 1.30 log_10_ CFU/cornea

*p < 0.05 compared with the vehicle (Williams test, one-sided)

### Effect of AZM on anterior ocular inflammation

**Fig. 2:**
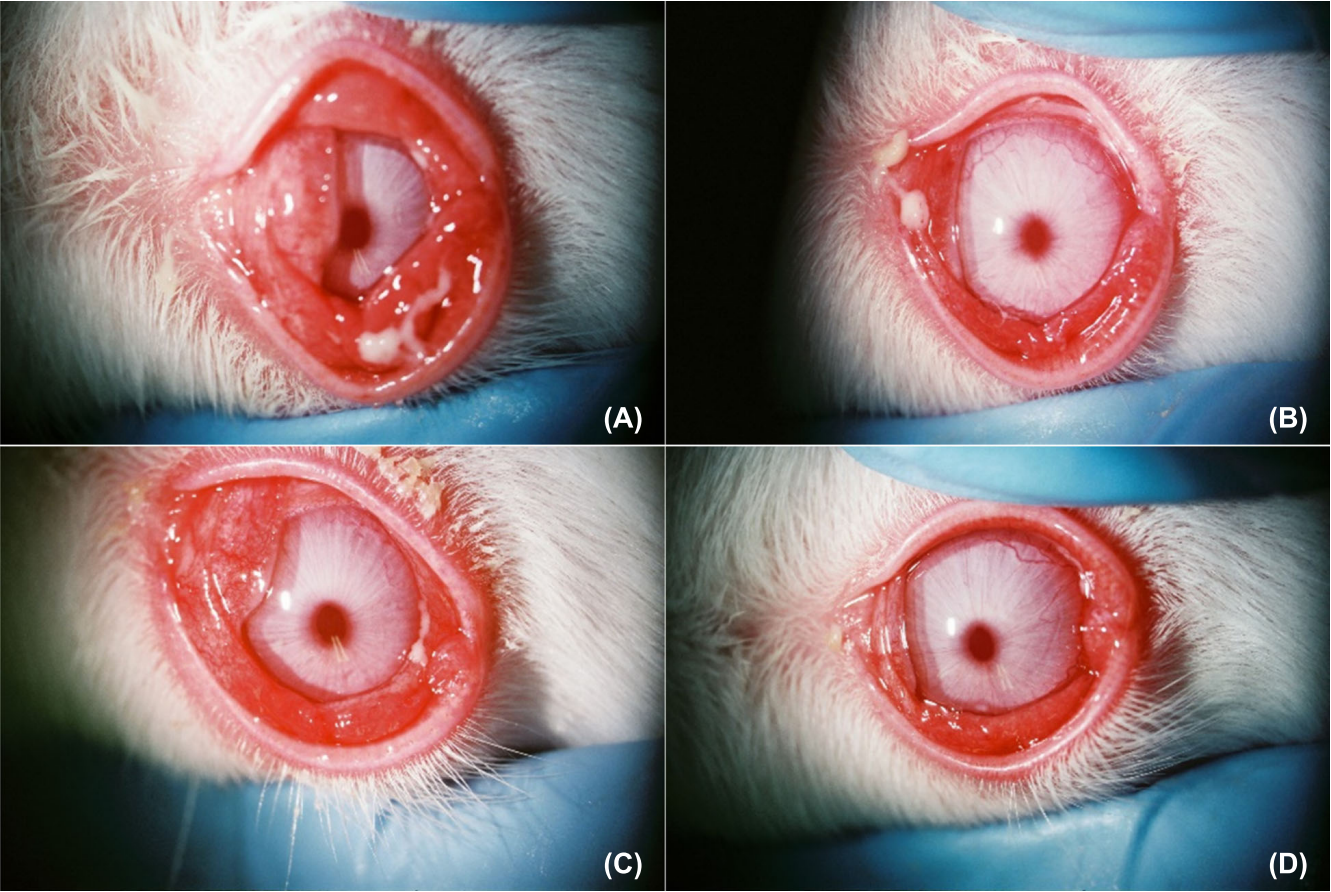
Bacteria culture supernatant induced anterior ocular inflammation in rabbits 12 h post induction. Conjunctival hyperemia and edema, nictitating membrane redness and edema, iris hyperemia, and ocular discharge were observed in the saline- (**a**), 0.1% betamethasone sodium phosphate- (**b**), and 0.1 and 1% azithromycin-treated eyes (**c**, **d**)

**Fig. 3:**
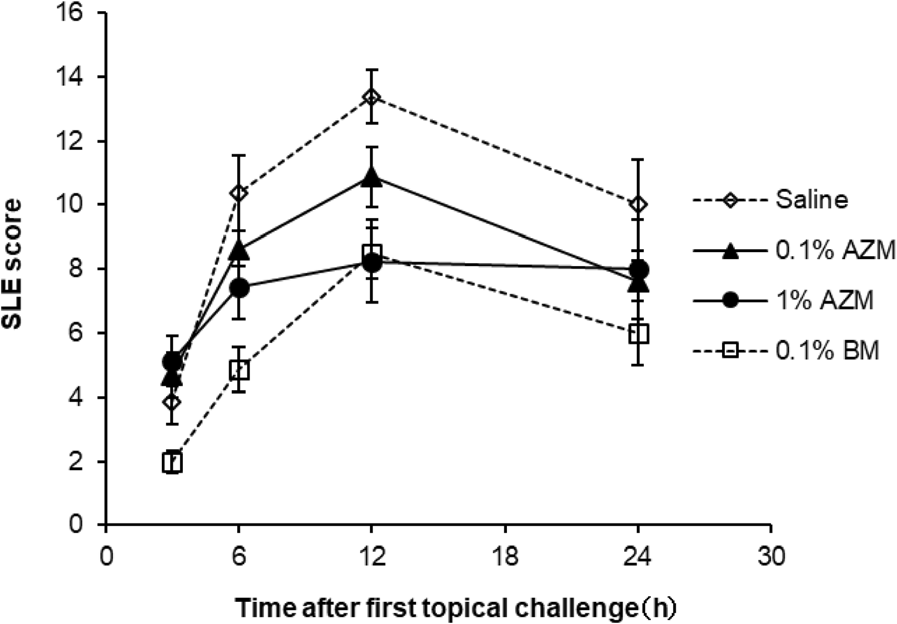
Effect of 0.1% azithromycin and 0.1% betamethasone sodium phosphate on aseptic anterior ocular inflammation induced by topical challenge with bacterial culture supernatant in rabbit eyes*.* The data represent the mean and standard error (*n* = 8)

Administration of SaS induced the clinical signs hyperemia and edema in the conjunctiva, redness and edema in the nictitating membrane, hyperemia in the iris, and ocular discharge (Fig. 2). In the saline group, the SLE score was increased at 3 h after induction and reached the maximum at 12 h after induction (Fig. 3, Table 3). The SLE scores of the AZM groups were lower than those of the saline group, and treatment with 1% AZM significantly reduced the SLE score at 12 h (*p* = 0.0113). The SLE score of the 0.1% betamethasone sodium phosphate group was significantly lower than that of the saline group at 6 and 12 h (*p* = 0.0085 and 0.0037, respectively). The SLE score of the 1% AZM group at 12 h was similar to that of the 0.1% betamethasone sodium phosphate group.

**Table 3:** Median of Slit lamp examination score in the rabbit anterior ocular inflammation model

Treatment		n	Median (1st quartile – 3rd quartile)			
			Time after first topical challenge			
			3 h	6 h	12 h	24 h
Saline		8	4.00 (2.25–5.13)	12.00 (8.38–12.25)	14.00 (12.00–14.25)	9.75 (7.25–12.25)
Azithromycin	0.1%	8	4.25 (3.50–5.63)	8.00 (7.50–9.00)	11.50 (9.75–12.38)	8.00 (7.50–8.63)
	1%	8	5.25 (4.00–6.25)	6.50 (5.38–10.25)	8.25 * (5.25–11.25)	7.75 (4.63–11.50)
Betamethasone phosphate ester	0.1%	8	2.00 (1.50–2.13)	5.25 ** (3.63–6.00)	8.75 ** (7.88–9.63)	6.00 (4.63–8.25)

*p < 0.05 and **p < 0.01 compared with the saline (Steel test, one-sided)

### Hemolytic reaction and secretion proteins in the bacterial supernatant

**Fig. 4:**
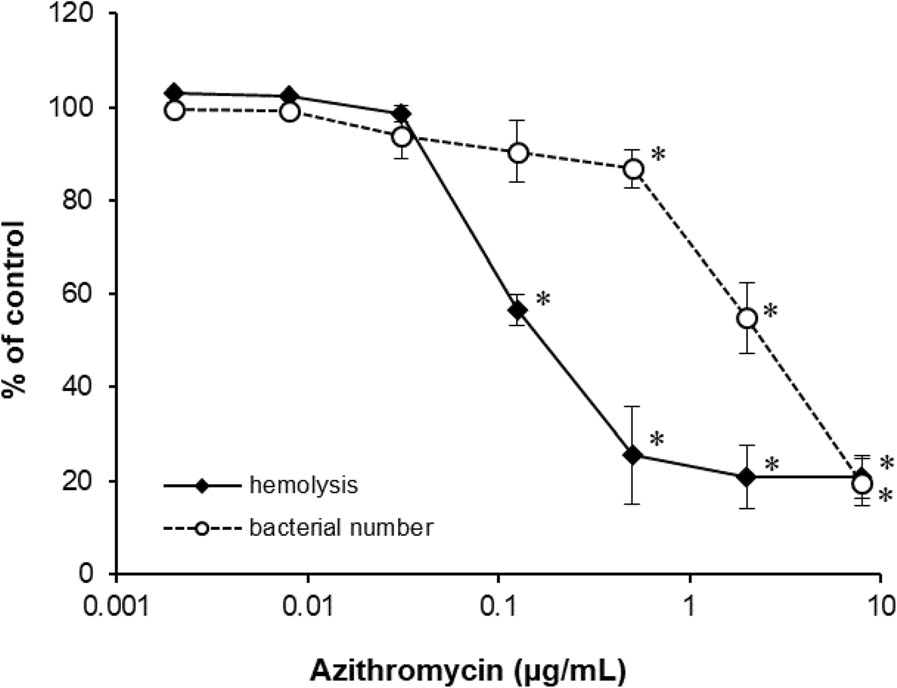
Inhibitory effect of azithromycin on production of bacterial virulence factor and bacterial growth. Experiments were performed in duplicate. Bacterial cells were grown in broth medium with increasing doses of azithromycin for 16 h. Rate of bacterial number density and hemolytic activity to control groups are shown. The data represent the mean and standard error (*n* = 3). The significance of differences compared with the control group is designated as *p < 0.05 (Williams test, one-sided)

Bacterial growth was significantly inhibited by AZM at or above 0.5 μg/mL concentration (Fig. 4). Hemolytic activity was significantly inhibited by AZM at or above 0.125 μg/mL concentration.

## Discussion

The objective of this study was to clarify whether the anti-inflammatory and anti-virulence effects of AZM contributed to its therapeutic effects against ocular infection. It is well known that macrolide exerts anti-inflammatory effects on the host, which was first reported by long-term treatment of respiratory diseases with erythromycin [20]. Although there are few reports of such effects in ophthalmology, the inhibitory effect of AZM on the production of proinflammatory cytokines has been demonstrated in human corneal epithelial cells [9]. Furthermore, it has been reported that the clinical signs in the conjunctiva were suppressed by topical application of AZM in acute anterior ocular inflammation models [15, 21]. However, the contribution of anti-inflammatory effects of AZM in the treatment of ocular infection has not been fully elucidated.

In the bacterial keratitis model of the present study, 1% AZM suppressed the clinical signs of ocular infection and inhibited bacterial growth in the cornea. On the other hand, 0.01 and 0.1% AZM suppressed the clinical signs but did not inhibit bacterial growth in the cornea. These data suggest the involvement of effects other than the anti-bacterial activity in the therapeutic effects of AZM against ocular infectious diseases. To elucidate these effects of AZM in a bacterial keratitis model, in the present study, we developed an SaS-induced anterior ocular inflammation rabbit model that represents the inflammation of ocular infections under bacteria-free conditions. *Staphylococcus aureus*-derived secretions including toxins mediate the destruction of ocular tissue and contribute to the induction of an inflammatory response [22–24]. Hemolytic toxin, such as alpha-toxin and Panton–Valentine toxin are thought to contribute to pathogenesis of ocular infection [5, 25, 26]. This kind of bacterial pore-forming toxins induces host cell lysis leading to destruction of corneal tissues in the infected eye. Instillation of SaS containing bacterial toxins, with confirmed hemolytic activity, caused inflammation in the anterior ocular surface. Therefore, our model partially represents the inflammation caused by ocular surface infection. In this model, 0.1 and 1% AZM suppressed the clinical signs under the same frequency of application as that in the rabbit bacterial keratitis model. These results reveal that AZM suppresses the clinical signs of ocular infection induced by bacterial virulence, and the mechanism is independent of the anti-bacterial effect. Conjunctival homogenate myeloperoxidase activity was examined in order to further confirm anti-inflammatory effects of AZM in this model, but that was undetectable in all groups (data not shown).

It is known that AZM at sub-minimal inhibitory concentration inhibits the production of bacterial virulence factor and formation of biofilm [11–14]. Bacterial secretion is one of the main causes of inflammation in the ocular surface, indicating that inhibiting the production of bacterial virulence factor could be effective in the treatment of ocular infection. Azithromycin decreased the hemolytic activity of *S. aureus* at a concentration that did not inhibit their growth. This result is associated with the therapeutic effect of AZM at the concentration of 0.1% or less in the rabbit bacterial keratitis model. .

There are some possible limitations to this study. First, because it was difficult to distinguish between the anti-bacterial effects and other effects of 1% AZM, we could not fully elucidate the extent to which those other effects contributed to the treatment of ocular infections. It may be possible to clarify this by comparing the treatment effects of appropriate anti-bacterial agents that only possess an anti-bacterial effect (without anti-inflammatory effect and anti-virulence effect) with that of 1% AZM on ocular infection. Furthermore, the effects of AZM on azithromycin-resistant bacteria may reveal the action of anti-inflammatory and anti-virulence effects. Secondly, although we tried to detect the bacterial toxins in the culture supernatant that were reduced by AZM using an immunoassay (data not shown), we could not identify these toxins. It is considered that AZM can suppress the production of pore-forming toxins, such as alpha-toxin and Panton–Valentine leucocidin, which are major virulence factors leading to corneal damage by *S. aureus* [5, 25–27]. The inhibition of bacterial virulence factors by AZM was not confirmed in vivo because the bacterial virulence factor influenced by AZM had not been identified in the in vitro study. It remains uncertain how the anti-virulence effect of AZM, which was shown to be independent of its anti-bacterial effect in this study, contributed to the treatment of ocular infections in vivo. Further studies are needed to understand the specific anti-virulence mechanism of AZM. Such studies can expand our understanding of the effects of AZM beyond that of its anti-bacterial effect and might provide new therapeutic strategies to prevent the emergence of resistant bacteria for ocular infections.

## Conclusions

The present study demonstrated that AZM exerts not only anti-bacterial activity but also anti-inflammatory effects on the host and inhibits the production of bacterial virulence factor. The results also elucidated that non-antibacterial effects contributed to the therapeutic effect of AZM against ocular infection. These effects of AZM potentially suppress ocular tissue damage from excessive inflammatory response, and consequently the therapeutic effect of AZM against ocular infection may be higher than that predicted from the in vitro anti-bacterial activity.

## Data Availability

The datasets used and/or analyzed during the current study are available from the corresponding author on reasonable request.

## Abbreviations

AZM: Azithromycin
CFU: colony-forming units
SaS: *Staphylococcus aureus* supernatant
SCD: soybean casein digest
SLE: Slit-lamp examination
TSB: tryptic soy broth
